# Design principles of the paradoxical feedback between pancreatic alpha and beta cells

**DOI:** 10.1038/s41598-018-29084-4

**Published:** 2018-07-16

**Authors:** Immacolata Garzilli, Shalev Itzkovitz

**Affiliations:** Weizmann Institute of Science, Department of Molecular Cell Biology, Wolfson Building, 234 Herzl St., Rehovot, 76100 Israel

## Abstract

Mammalian glucose homeostasis is controlled by the antagonistic hormones insulin and glucagon, secreted by pancreatic beta and alpha cells respectively. These two cell types are adjacently located in the islets of Langerhans and affect each others’ secretions in a paradoxical manner: while insulin inhibits glucagon secretion from alpha cells, glucagon seems to stimulate insulin secretion from beta cells. Here we ask what are the design principles of this negative feedback loop. We systematically simulate the dynamics of all possible islet inter-cellular connectivity patterns and analyze different performance criteria. We find that the observed circuit dampens overshoots of blood glucose levels after reversion of glucose drops. This feature is related to the temporal delay in the rise of insulin concentrations in peripheral tissues, compared to the immediate hormone action on the liver. In addition, we find that the circuit facilitates coordinate secretion of both hormones in response to protein meals. Our study highlights the advantages of a paradoxical paracrine feedback loop in maintaining metabolic homeostasis.

## Introduction

Homeostasis is a specialized form of regulation that precisely maintains the function of a system at a set point^1^. It is a hallmark of mammalian physiology: temperature, pH, fluid volume, calcium levels and blood pressure are some examples of quantities in the body that are maintained at constant levels. Blood glucose levels are maintained at approximately 5 *mM* in humans (90 *mg*/*dL*)^2^ and rarely exceed 6.9 *mM* or drop below 3.8 *mM*^3,4^.

**Figure 1:**
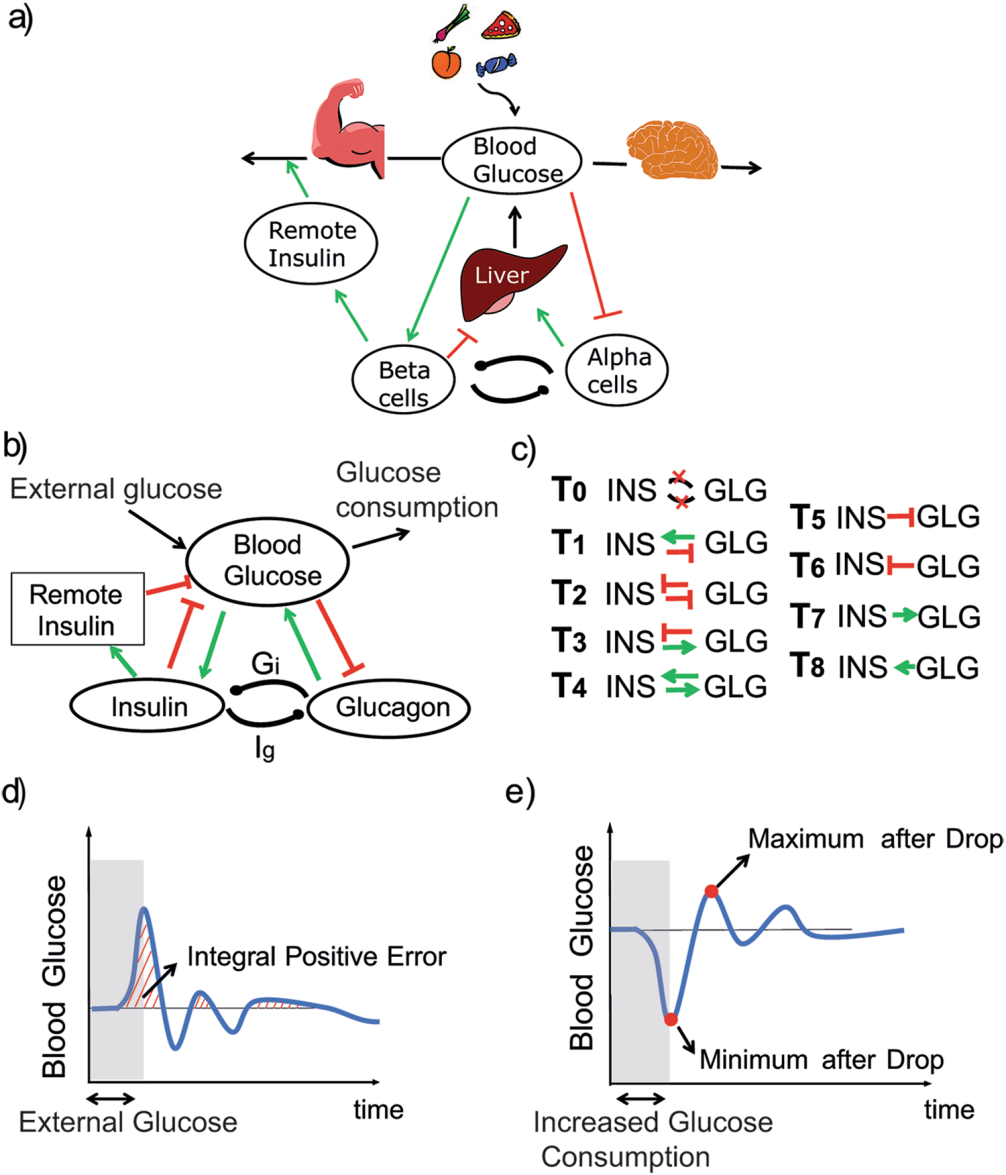
The intra-islet network and alpha and beta cell interactions. (**a**) Scheme for the blood glucose homeostasis mechanism: alpha and beta cells mediate two negative feedback loops with glucose through the liver’s glucose production. Remote insulin promotes the uptake of glucose from the blood by muscles and bones, thus decreasing its level; brain asserts a constant blood glucose consumption; food represents an external source of glucose. (**b**) Scheme for the intra-islet topology: green arrows represent activating interactions, red arrows are inhibitory interactions. Insulin and glucagon affect each other's secretion with strengths *I*_*g*_ and *G*_*i*_ respectively; external glucose represents meals, glucose consumption represents exercise. (**c**) All possible combinations of paracrine interactions: topology *T*_0_ has no direct interaction between the hormones (*I*_*g*_ = *G*_*i*_ = 0); *T*_1_ − *T*_4_ are topologies with both nonzero interactions (*I*_*g*_ ≠ 0 and *G*_*i*_ ≠ 0); *T*_5_ − *T*_8_ are topologies with only one nonzero interaction (*I*_*g*_ ≠ 0 or *G*_*i*_ ≠ 0); (**d**) Blood glucose response to external glucose; the deviation from the steady state value is scored by the Integral Positive Error; (**e**) Blood glucose response to an increase in systemic glucose consumption; the deviation from the steady state value is scored by the minimum level after the drop and the first maximum overshoot after glucose reversion to steady state.

Glucose homeostasis is controlled by two antagonistic hormones, insulin and glucagon, secreted by beta and alpha cells respectively. These two cell types are adjacently located in the islet of Langerhans^5^. Insulin is secreted by beta cells in response to elevated blood glucose levels (i.e. meals) and brings about an immediate cessation of glucose production by the liver and a systemic uptake of glucose for storage by tissues such as the liver and muscle, thus lowering glucose to its basal state. In contrast, glucagon instructs the liver to rapidly release glucose into the circulation when plasma glucose levels are low (i.e. fasting or exercise, Fig. 1a). Thus, insulin and glucagon actions on blood glucose levels mediate two negative feedback loops in which insulin acts as a repressor, while glucagon as activator (Fig. 1b). Failure of beta cells to secrete insulin in diabetic patients results in uncontrolled fluctuations in blood glucose levels. In addition to their action on blood glucose levels, glucagon and insulin are jointly secreted in response to protein intake^6,7^. This correlated secretion stems from the additional role of insulin as a stimulator of cellular consumption of metabolized amino acids. Co-secretion of glucagon under protein-rich, carbohydrate-poor meals is thought to counteract the simultaneous effects of insulin on blood glucose levels, thus preventing dangerous glucose drops termed “hypoglycemia”^8^. Although islet architecture, glucose response to external stimuli and, in general, metabolism, are different from species to species^9–15^, the described core mechanism is considered to be common to most mammals.

Extensive evidence suggests that alpha and beta cells do not operate independently in regulating blood glucose levels. Rather, they both sense and modulate each other's secretions. Alpha and beta cells are spatially adjacent in the islets of Langerhans (to different extent in human and mouse) and each cell type expresses the receptors for its antagonistic hormone. Since intra-islet hormone concentrations are 100 times higher than those in circulation^16^, this islet architecture could efficiently couple alpha and beta cells. A natural circuitry to implement the antagonistic secretion of insulin and glucagon would be a mutual inhibition (*T*_2_ topology, Fig. 1c)^17^. However, this does not seem to be the case for the intra-islet interactions. Whereas insulin secretion has been shown to repress glucagon secretion^18–22^, glucagon in turn stimulates insulin secretion^23–26^. This stimulation seems paradoxical when considering blood glucose levels, as low glucose levels would directly inhibit insulin secretion, yet indirectly stimulate it through its induction of glucagon (*T*_1_ topology, Fig. 1b,c).

Previous mathematical models of glucose homeostasis focused on the action of insulin on blood glucose levels^17,27–32^. Other models considered the effect of coupling between alpha and beta cells on the relative phases of insulin and glucagon pulsatile secretion^33–35^. Jo *et al*.^26^ highlighted an advantage for the observed negative feedback circuit in minimizing blood glucose deviations following glucose stimulations. Here we ask what might be the functional advantage of the paradoxical feedback between alpha and beta cells in maintaining homeostasis under diverse stimulations, including glucose steps and drops, as well as amino acid meals. We use modelling and simulations to systematically analyze the performance of all possible intra-islet circuits consisting of alpha and beta cells and examine their ability to minimize temporal deviations of glucose from baseline levels in face of external perturbations that model meals and exercise. We demonstrate that the feedback minimizes transient overshoots in response to glucose steps or drops, and show that it has advantageous features in co-secretion in response to protein meals.

## Results

### Mathematical model of the islet circuit

To characterize the circuit underlying the control of blood glucose, we derived a mathematical model of 4 Ordinary Differential Equations (ODEs) that took into account experimental observations and hypotheses from literature. For simplicity, we neglected the effect of Somatostatin, a hormone secreted by the islets' delta cells in response to insulin secretion and which inhibits the secretion of both insulin and glucagon. Our model describes the rate of changes of the concentration of the following quantities over time: Blood glucose ([*BG*]), blood glucagon ([*GLG*]), blood insulin ([*INS*]) and remote insulin ([*Rins*]), an intermediate factor representing insulin concentrations in the interstitial tissue compartment. [*Rins*] is considered to mediate the delayed effect of the insulin repression on blood glucose^28^. We considered all the possible combinations of negative and positive interactions between insulin and glucagon, yielding nine different sub-models (Fig. 1c).

The following general ordinary differential equations describe the dynamics of all of the possible endocrine circuits:

1 $$\begin{array}{rcl}\frac{d[BG]}{dt} & = & INPUT+{\beta }_{0}f([GLG],[INS])-({\delta }_{b}+{\delta }_{b}DROP)[BG]-V[Rins][BG]\end{array}$$

2 $$\frac{d[GLG]}{dt}=\alpha -{\delta }_{g}[GLG]+{V}_{r}{(B{G}^{\ast }-[BG])}^{+}+{I}_{g}g([INS])$$

3 $$\begin{array}{rcl}\frac{d[INS]}{dt} & = & \mu +K{([BG]-B{G}^{\ast })}^{+}-{\delta }_{i}[INS]-\varepsilon ([INS]-[Rins])+\,{G}_{i}h([GLG])\end{array}$$

4 $$\frac{d[Rins]}{dt}=\varepsilon ([INS]-[Rins])-{\delta }_{Ri}[Rins].$$

*INPUT* represents glucose uptake from a meal, *DROP* represents an increase in blood glucose systemic uptake, for example due to increased muscle consumption during exercise. (*x*)^+^ is 0 when *x* ≤ 0 and *x* when *x* > 0. At steady state, in absence of external perturbation, *INPUT* = *DROP* = 0. We simulated the dynamic response of the endocrine circuits to external perturbations by changing the variables INPUT and DROP (Fig. 1d,e). *f*([*GLG*], [*INS*]) represents the hepatic glucose output (HGO) as a function of the blood glucose levels of [*INS*] and [*GLG*]^36,37^. We considered the following form consisting of a sum of two Michelis Menten (MM) terms:

5 $$f([GLG],[INS])=(\omega \frac{[GLG]}{GL{G}^{\ast }+[GLG]}+(2-\omega )\frac{IN{S}^{\ast }}{IN{S}^{\ast }+[INS]})$$

GLG^*^ and INS^*^ represent the steady states of glucagon and insulin at [*BG*] = 5 *mM*, and 0 ≤ *ω* ≤ 2 represents the relative weight attributed to glucagon over insulin in affecting the liver’s response.

For each equation we considered degradation terms for glucagon, insulin and remote insulin (*δ*_*g*_, *δ*_*i*_, *δ*_*Ri*_ respectively); *δ*_*b*_ represents basal blood glucose uptake, predominantly through brain consumption and *V* represents the insulin dependent glucose uptake^27^; *α* and *μ* represent the basal secretion rates of glucagon and insulin. We modelled insulin secretion as a monotonically increasing function of blood glucose levels, *K*([*BG*] − BG^*^)^+^, and glucagon secretion as a monotonically decreasing function of blood glucose levels *V*_*r*_(BG^*^ − [*BG*])^+^ ^38^. The equations for insulin (3) and remote insulin (4) have a common “transport term” (*ε* ([*INS*] − [*Rins*])) representing insulin diffusion from the blood to the interstitial compartment^28^.

Interactions between insulin and glucagon are expressed by two generic functions *I*_*g*_*g*([*INS*]) and *G*_*i*_*h*([*GLG*]). For simplicity, in the following analysis, *g*([*INS*]) and *h*([*GLG*]) will be linear, but the results remain valid for nonlinear functions(Supplementary Information). Our model also ignores direct effects of blood insulin on increasing liver glucose consumption via glycogenesis. As with non-linearities, the results below remain valid when introducing this additional process (Supplementary Information). The model in Equations (1–5) has 16 free parameters, which we next reduced to 5 parameters (*I*_*g*_, *G*_*i*_, *V*_*r*_, *K*, *ω*) using different estimates from literature (Table 1).

**Table 1:** Table of estimated parameters: parameters *δ*_*b*_, *δ*_*g*_, *δ*_*i*_, *δ*_*ri*_, *V*, BG^*^, INS^*^, GLG^*^ are estimated from literature; parameters *β*_0_, *α*, *ε*, RINS^*^ are evaluated from the steady state conditions in Equations (1–4), see Methods. Parameters μ, Vr, K, w are estimated as described in Methods

Parameter	Values	Units	Meaning	Reference
*β*_0_		*pmol* × *min*^−1^/*L*	HGO (constant)	steady state
*μ*	62	*pmol* × *min*^−1^/*L*	insulin basal production	estimated
*α*		*pmol* × *min*^−1^/*L*	glucagon basal production	steady state
*δ*_*b*_	0.026	*min* ^−1^	glucose degradation	see^27^
*δ*_*g*_	0.1155	*min* ^−1^	glucagon degradation	see^59^
*δ*_*i*_	0.2	*min* ^−1^	insulin degradation	see^60^
*δ*_*ri*_	0.01	*min* ^−1^	remote insulin degradation	see^28^
*V*	0.38 × 10^−6^	*pmol*^−1^ *min*^−1^ *L*	remote insulin action	see^27^
*V*_*r*_	[10^−7^, 10^−3^]	*min* ^−1^	effect of glucose on glucagon	estimated
*K*	[10^−7^, 10^−3^]	*min* ^−1^	effect of glucose on insulin	estimated
*ε*		*min* ^−1^	remote insulin diffusion	steady state
*I*_*g*_	[−0.5, 0.5]	*min* ^−1^	Insulin direct action on Glucagon	hypothesis
*G*_*i*_	[−0.5, 0.5]	*min* ^−1^	Glucagon direct action on Insulin	hypothesis
*INPUT*	1.4 × 10^8^	*pmol* × *min*^−1^/*L*	external pulse	hypothesis
*DROP*	2	*min* ^−1^	external drop	hypothesis
*BG* ^*^	5	*mM*	blood glucose steady state	see^4^
*INS* ^*^	174	*pmol*/*L*	insulin steady state	see^58^
*GLG* ^*^	17.2	*pmol*/*L*	glucagon steady state	see^59^
*RINS* ^*^		*pmol*/*L*	remote insulin in plasma	steady state
*ω*	[0, 2]		glucagon steady state	estimated

.

### Performance criteria

We considered three different performance criteria for the endocrine systems studied. The first was a low integral positive error, defined as:

6 $${\int }_{0}^{T}{([BG]-B{G}^{\ast })}^{+}dt$$

where [0, *T*] represents the interval in which the simulation is performed.

This criterion represents the ability of a circuit to avoid hyperglycemia following a glucose step (Fig. 1d). The second criterion was a high value for the minimum glucose level in response to increased systemic glucose consumption, thus avoiding large hypoglycemic glucose drops (Fig. 1e). The third was a low overshoot when blood glucose reverts from a hypoglycemic state (Maximum after drop, Fig. 1e).

### Response to glucose perturbations - local analysis

**Figure 2:**
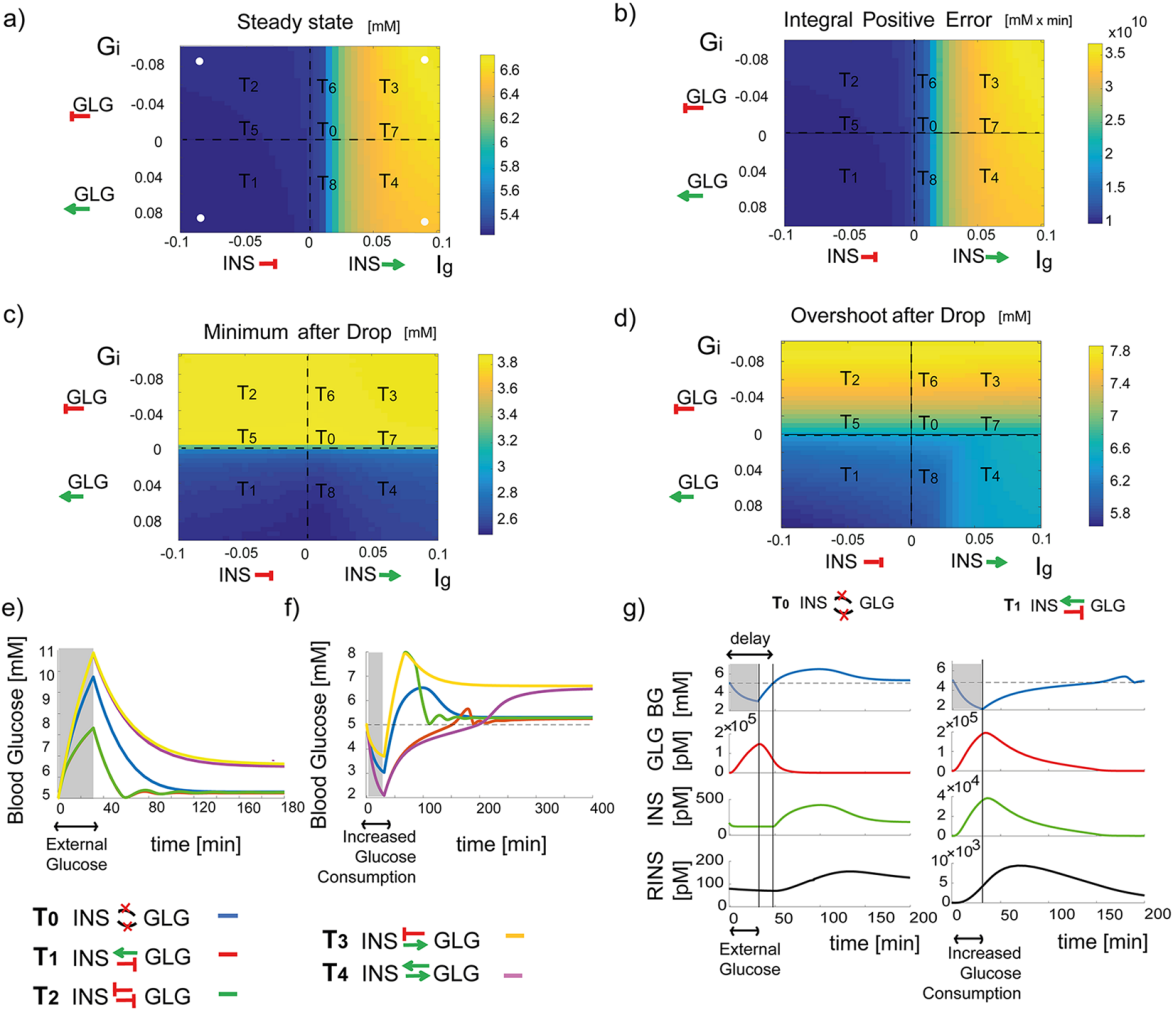
Local analysis reveals features of all circuit topologies. Shown are the blood glucose steady states (**a**) integral positive error (**b**) minimum level after a glucose drop (**c**) and maximum overshoot after reversion from glucose drops (**d**). For each pair of parameters (*I*_*g*_, *G*_*i*_) the system responses to 30 minute glucose *INPUT* and 30 minute glucose *DROP* were simulated and scored. Integral error has been evaluated on a 500-minute time interval. White dots represent each pair of parameters represented in (**e** and **f**); different areas are labeled with the corresponding topology. (**e-f**) Simulations of blood glucose response to a 30-minutes positive/negative pulse: external stimuli are represented by the grey shaded area and response of systems *T*_0_, *T*_1_, *T*_2_, *T*_3_, *T*_4_ are represented respectively in blue, red, green, yellow and purple. (**g**) Comparison between topologies *T*_0_, *T*_1_, for the behavior of blood glucose ([*BG*]), glucagon ([*GLG*]), insulin ([*INS*]) and remote insulin ([*Rins*]), after an increased glucose consumption of 30 minutes (grey shaded area): in the *T*_0_ topology insulin ramps up with a delay of 50 minutes. In all the analyses, *V*_*r*_ = *K* = 10^−5^ and *ω* = 1.

To understand the potential utility of the paradoxical negative feedback loop between alpha and beta cells we next applied a strategy that we term “local analysis”. We stimulated the system without paracrine interaction, *T*_0_ (Fig. 1c), with a positive or negative 30-minute step of *INPUT* and *DROP* (Fig. 2e,g). We scanned the 3-dimensional parameter space of *T*_0_ (*V*_*r*_, *K*, *ω*) and identified a combination that leads to a relatively favorable performance in terms of the three criteria (the following results are insensitive to the *T*_0_ parameters). Next, we systematically modified the strengths and directions of the paracrine interactions *G*_*i*_ and *I*_*g*_ and assessed the effects on the system performance criteria (Fig. 2a–d).

We found that the circuit topologies *T*_1_, *T*_2_ and *T*_5_ were better than others in minimizing the integral positive error. These circuits include inhibition of glucagon secretion by insulin, thus ensuring efficient shut-down of hepatic glucose output upon feeding. Thus, upon an increase in glucose levels, glucagon levels would decrease both directly by glucose as well as indirectly by the increase in insulin secretion (Fig. 2b). Topologies *T*_3_, *T*_4_ and *T*_7_ fared much worse in terms of integral error as they included indirect activation of glucagon by insulin in parallel to its direct inhibition by glucose. Moreover, they could not achieve the 5 *mM* steady state for a broad range of parameters, as shown in Fig. 2a.

When considering the response to glucose drops, we found that *T*_1_, *T*_4_ and *T*_8_ displayed a lower minimum, a result of the paradoxical activation of insulin secretion by glucagon (Fig. 2c). Notably, however, these topologies gave rise to a significantly reduced overshoot upon reversion of glucose levels to its steady state, as evident by the lower maximum glucose levels upon reversion of glucose drops (Fig. 2d). This unique feature of *T*_1_, *T*_4_ and *T*_8_ is related to the time scales involved. Equation (1) considers that blood glucose levels are reduced systemically through the action of remote insulin, [*Rins*]. However, [*Rins*] rises at a delay compared to insulin due to its diffusion from the blood to the interstitial compartments (Fig. 2g). Following reversion of blood glucose levels after a 30-minutes hypoglycemic drop, if insulin secretion would increase only when [*BG*] levels exceeded 5 *mM* there would be a delay of 50 minutes until [*Rins*] will have ramped up to the required level necessary to reduce [*BG*] levels (Fig. 2g). The paradoxical activation of insulin secretion by glucagon produces an early rise in [*Rins*], so that its systemic levels are high enough once [*BG*] reaches 5 *mM* to blunt additional overshoots (Fig. 2g).The ability of the system to counteract an overshoot following reversion of blood glucose level drops is valid for a wide range of delay values, Supplementary Fig. S1.

Thus, *T*_1_, the circuit observed in the islets, seems to lead to a lower minimum blood glucose level compared to other topologies but has two attractive features in terms of glucose homeostasis - minimizing the integral positive error in response to glucose input and blunting the overshoot of glucose levels following reversion of hypoglycemia. We will demonstrate below that the potentially dangerous undershoots associated with this topology can be minimized by modulating the liver input function to insulin and glucagon.

### Response to glucose perturbations - global analysis

**Figure 3:**
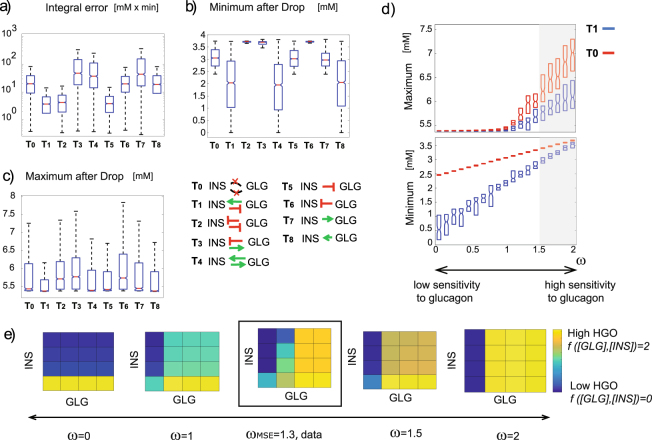
Global analysis reveals features of all circuit topologies. (**a**) Boxplots represent the integral positive error (**a**) minimum level after drop (**b**) and maximum overshoot after drop (**c**) of all circuit topologies. For each index, Kruskal-Wallis analysis reports *pvalue* < 0.001. (**d**) Undershoot of circuit *T*_1_ can be minimized by increasing the liver sensitivity to glucagon. Grey shaded area represents the range of *ω* for which minimum is higher than 3 *mM*. (**e**) Hepatic glucose output as a function of glucagon and insulin is shown depending on the value of the liver sensitivity to glucagon (parameter *ω*). Simulations have been performed considering Equation (5) as input function with parameters reported in Table 1. Plot in the black rectangle represents the data obtained from^37^. GLG ∈ [0, 5000] *pM* and INS ∈ [0, 10000] *pM*. GLG and INS axis are shown in logarithmic scales.

Our local analysis considered the system’s performances when only the two-paracrine strength were varied and all the other parameters remained fixed. To complement this analysis we performed an unbiased numerical screen^39–41^ by sampling parameters at random from the 5-dimensional parameter space, consisting of (*V*_*r*_, *K*, *ω*, *I*_*g*_, *G*_*i*_) and scoring the resulting topologies (Figs 3 and S2). Here again, we found that topologies *T*_1_, *T*_2_ and *T*_5_ were better at minimizing the integral positive error in response to a glucose input. This improved performance was born out of the inhibition of glucagon secretion by both glucose as well as insulin (Fig. 3a). *T*_1_, *T*_4_ and *T*_8_, the topologies in which glucagon activates insulin, led to lower glucose levels in response to increased glucose consumption (Fig. 3b), but were much better in avoiding overshoots following reversion to normal [*BG*] levels (Fig. 3c). Similar results are shown for the case in which one of the paracrine interactions or both are modeled as nonlinear (Supplementary Fig. S3) or when a term for glycogenesis is considered in Eq. (1), Supplementary Fig. S4.

### The liver's input function to glucagon and insulin can minimize undershoots during hypoglycemia

Our analysis indicates that the paradoxical stimulation of insulin secretion by glucagon minimizes overshoots in blood glucose levels following reversion of hypoglycemia, but has a notable vulnerability - glucose levels drops are accentuated (low minimum after drops, Figs 2 and 3). Since hypoglycemic events of low blood glucose levels may be life threatening we examined whether this trade off could be alleviated by modulating the combined effects of glucagon and insulin on the hepatic glucose output. We found that an input function *f*([*GLG*], [*INS*]) (Equation (5)) in which the liver is more sensitive to glucagon compared to insulin (*ω* > 1) facilitates a low overshoot as well as a reduced undershoot (Figs 3d and S5). With such an input function, insulin is less effective in shutting down the hepatic glucose output. Previous data indicate that such increased sensitivity in which glucagon ‘over-rides’ the signal from insulin is indeed observed^37^, Fig. 3e. To assess the *ω* parameter that best describes the previously measured liver input function to insulin and glucagon, we scanned a range of 0 ≤ *ω* ≤ 2 and computed the Mean Squared Error (MSE) between the experimental and theoretical input functions, both maximized to their maximal value. We obtained *ω*_*MSE*_ = 1.3.

### Response to protein meals

Our previous analysis demonstrated that the inhibition of glucagon secretion by insulin gives rise to a decrease in the integrated positive error following an *INPUT* glucose meal. Moreover, we found that the paradoxical stimulation of insulin secretion by glucagon minimizes overshoots of [*BG*] levels when reverting from a hypoglycemic step, at the expense of a lower minimum after drop which can be prevented by the liver input function *f*([*GLG*], [*INS*]). We next considered additional possible advantages of the paradoxical topology *T*_1_ over alternative more intuitive topologies such as *T*_2_, in which insulin and glucagon mutually repress the secretion of their cognate hormones. To this end, we analyzed the response of the circuit to protein meals.

**Figure 4:**
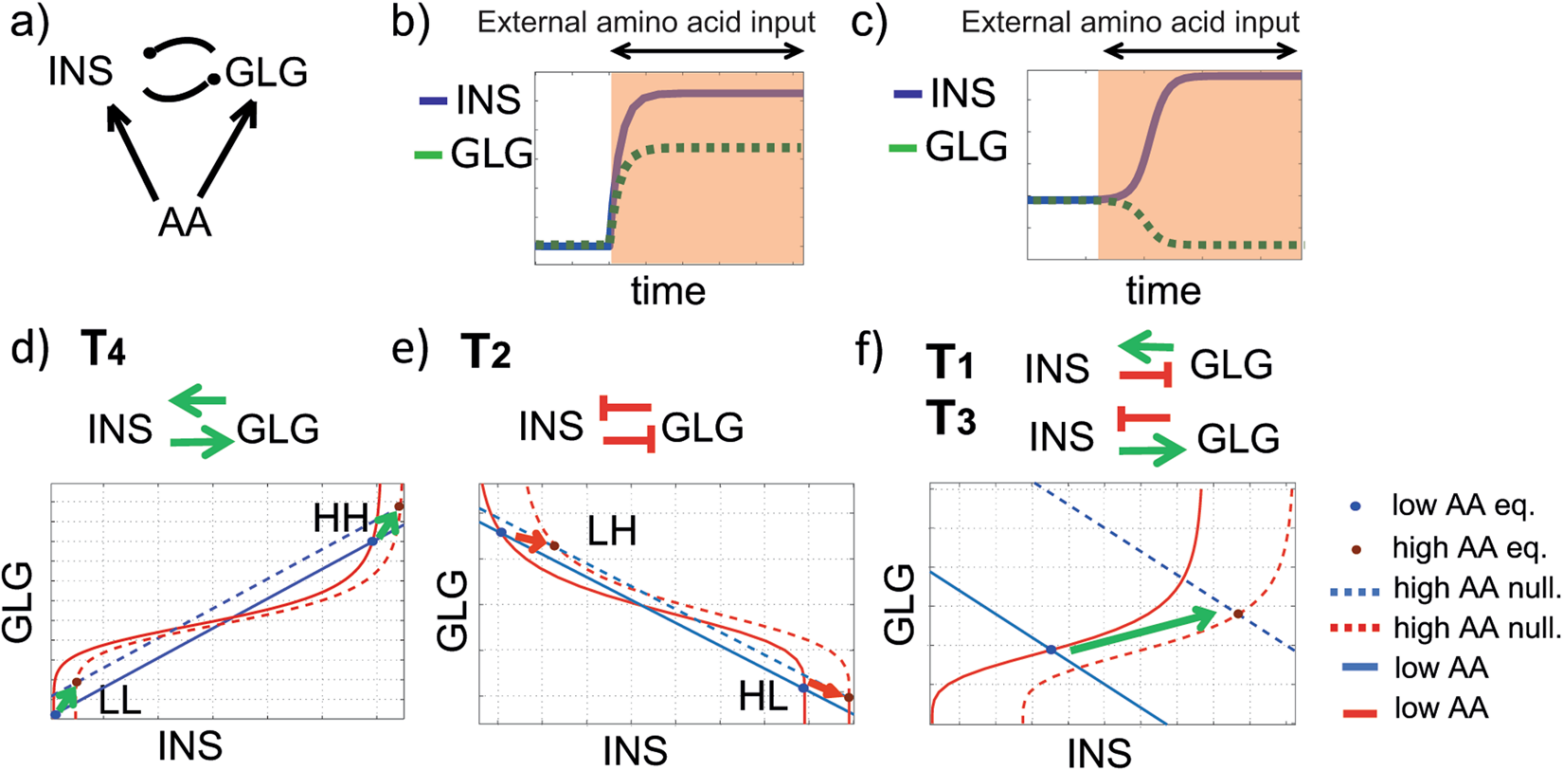
Circuit responses to amino acid inputs. (**a**) Amino acids (AA) stimulate the secretion of both insulin and glucagon. (**b**) Schematic example of coordinated behavior of insulin (blue line) and glucagon (green dashed line) over time after a step amino-acid stimulus (orange shaded area). (**c**) Schematic example of uncoordinated behavior of insulin (blue line) and glucagon (green dashed line) over time after a step amino-acid stimulus (red shaded area). (**d**) Schematics of nullclines for the *T*_4_ circuit with a double positive interaction between the two hormones, without AA input (continuous lines) and with the AA input (dashed lines): intersections (dots) represent steady states (LL - HH). (**e**) Schematics of nullclines for the *T*_2_ circuit with a double negative interaction between the two hormones without AA input (continuous lines) and with the AA input (dashed lines); intersections (dots) represent steady states (LH - HL). After the AA stimulus, nullclines shift and both steady states decrease (red dots). (**f**) Schematics nullclines for a negative feedback between the hormones (continuous lines); the intersection (dot) represents a single steady state; after the AA stimulus, nullclines shift (dashed lines) and both hormones increase (HH).

Unlike glucose, amino acids elicit a potent coordinated secretion of both insulin and glucagon. This coordination stems from the multiple roles of insulin as an anabolic hormone. Insulin is required not only to increase cellular uptake of glucose but also to increase lipogenesis in response to lipid intake and protein production via translation in response to consumption of amino acids^42,43^. Indeed, arginine is a more potent secretagogue of insulin than glucose^44^. The increased secretion of insulin in response to protein meals could have a dangerous impact on blood glucose homeostasis, yielding an insulin-dependent increase in [*BG*] consumption and a decrease in HGO. To counteract this decrease in blood glucose level, glucagon is also potently stimulated by amino acids (AA)^42,45–51^ to ensure increased HGO in face of such collateral blood glucose uptake (Fig. 4a).

To assess the potential of different islet circuits for co-secretion of both insulin and glucagon, we examined the steady state levels of insulin and glucagon in response to an intake of amino acids (AA). We used Equations (2 and 3) but instead of a glucose stimulation, we added constant terms *I*_*AAg*_ and *I*_*AAi*_ describing the amino acid stimulus on the secretion of insulin and glucagon (Fig. 4a):

7 $$\frac{d[GLG]}{dt}=\alpha +{I}_{AAg}-{\delta }_{g}[GLG]+{I}_{g}g([INS])$$

8 $$\frac{d[INS]}{dt}=\mu +{I}_{AAi}-{\delta }_{i}[INS]+{G}_{i}h([GLG])$$

where, [*GLG*] and [*INS*] are the two hormones, *α* and *μ* are the basal production rates for the two hormones, *I*_*AAg*_ = *I*_*AAg*_(*AA*) and *I*_*AAi*_ = *I*_*AAi*_(*AA*) are the production rates generated by the amino-acid input, *δ*_*g*_ and *δ*_*i*_ the degradation rates and *I*_*g*_*g*([*INS*]) and *G*_*i*_*h*([*GLG*]) are two general functions, representing the actions of one hormone on the other: in the double positive case *T*_4_ both *g*([*INS*]) and *h*([*GLG*]) are increasing, in the double negative case *T*_2_, they are both decreasing, whereas in the mixed cases *T*_1_ and *T*_3_ one is decreasing and the other is increasing.

In order to compare the performance of the different systems, we considered the constraints on *I*_*AAg*_, *I*_*AAi*_ that would ensure that the steady states of both hormones increase upon an input (*I*_*AAg*_, *I*_*AAi*_ > 0), see Methods.

We found that the negative feedback circuits *T*_1_ and *T*_3_ involving insulin and glucagon require a constraint only on one of the two pulses, while the double negative circuit *T*_2_ requires constraints on both the input functions *I*_*AAg*_, *I*_*AAi*_.

For simplicity, we consider one linear (h(GLG]) and one non-linear interactions (g[INS]), the results below are only valid when at least one interaction is non-linear). In particular:

9 $$g([INS])=\frac{IN{S}^{n}}{{K}^{n}+IN{S}^{n}}$$

in case of activation, or

10 $$g([INS])=\frac{{K}^{n}}{{K}^{n}+IN{S}^{n}}$$

in case of inhibition, with *n* > 2 and *K* > 0 and *h*([*GLG*]) = *G*_*i*_[*GLG*].

Figure 4d,e shows the nullclines for the double positive and the double negative cases respectively: in the double positive case, bistability is characterized by two equilibrium points, one in which both steady states are high (HH), and a second, in which both steady states are low (LL, Fig. 4d); in the double negative circuit *T*_2_, bistability is characterized by two equilibria in which one hormone is at a high steady state and the other is at a low steady state (HL, LH), giving rise to a switch or a “mutual inhibition” behavior Fig. 4e^52–55^. In the case of a negative feedback loop composed of one negative and one positive interaction (*T*_1_, *T*_3_) there is no bistability. Rather, in this case, the nullclines have only one intersection, i.e. a monostable behavior. Thus, a negative feedback completely avoids bistable behaviors and in particular opposite states of the hormones.

The simplified model considered above also demonstrates an example of how an intake of AA can lead to a coordinated higher secretion of both insulin and glucagon for *T*_1_, *T*_3_ and *T*_4_ but not *T*_2_ through the particular shift in the nullclines intersection points.

In summary, the paradoxical feedback *T*_1_ avoids bistability in response to AA, and is more robust in eliciting a coordinated secretion of hormones after an amino acid perturbation: coordination is possible with a restriction on the production rate of one of the hormones, while, more contraints are needed to have coordination with a double negative feedback system. We also assessed the impact of an amino acid meal, on blood glucose levels, by using Equations (1) and (4) in addition to (9 and 10). We found that when the liver is more sensitive to glucagon compared to insulin (parameter *ω* > 1, Fig. 3e) topologies *T*_1_, *T*_2_ and *T*_5_ are the best in minimizing the effect of an amino acid input on blood glucose levels in terms of integral deviation, as shown in Supplementary Fig. S6.

## Discussion

Alpha and beta cells implement a fundamental cell circuit that maintains glucose homeostasis. Glucagon and insulin are antagonistic in their action on blood glucose levels and thus expected to be mutually exclusive; insulin should be secreted when blood glucose levels are high, and glucagon when blood glucose levels are low. A classic circuit to implement such mutual secretion entails the mutual inhibition of secretion of each hormone by its antagonistic counterpart^17,52,53,56^. Surprisingly, while insulin indeed inhibits glucagon secretion, glucagon seems to stimulate insulin secretion. Our study aimed to understand the design principles underlying this paradoxical circuit. We found that the stimulation of insulin secretion by glucagon prevents overshoots of glucose levels when reverting from a hypoglycemic glucose drop. This feature is borne out the delay in the remote insulin action on the peripheral glucose uptake. Since remote insulin takes around 10 minutes to ramp up following an increase in beta cell insulin secretion, coordinated secretion of insulin, together with a rise in glucagon upon glucose drops, can facilitate the slow rise in remote insulin, in anticipation for the ensuing blood glucose rise. When blood glucose level reverts back to its steady state, remote insulin would already be high enough to blunt overshoots and stabilize blood glucose levels.

The ability of the paradoxical stimulation of beta cell insulin secretion by glucagon to blunt overshoots comes at a price of increased undershoots. Notably, we found that the liver response to the two hormones can minimize this effect. Unlike the systemic effect of insulin on all body tissues, glucagon predominantly affects the liver. By providing a precedence for glucagon over insulin in dictating the levels of HGO, the liver effectively ignores the mixed signals provided by the increase in insulin, so that insulin increase in response to hypoglycemia would serve to slowly ramp up remote insulin in anticipation of overshoots, while preventing cessation of HGO. Previous studies suggest that the liver input function indeed exhibits such increased sensitivity to glucagon over insulin^36,37^ (Fig. 3d,e).

In addition to the advantages conferred by the circuit in maintaining blood glucose homeostasis, we found that the paradoxical circuit facilitates coordinated secretion of glucagon and insulin in response to an amino-acid input. While insulin and glucagon are antagonistic in their function with respect to glucose, they are co-secreted in response to protein meals. We found that the negative circuit involving insulin and glucagon facilitates a monotonic increase in the levels of both hormones for a wider range of parameters. A non-paradoxical mutual inhibition between insulin and glucagon could also lead to bistability, an unwanted feature in a physiological control system such as the islet of Langerhans.

Our model ignored important features of the endocrine circuitry, including innervation, the effect of somatostatin^33,35^, incretins as well as stress hormones^57^. Beside the action of glucose, beta cells also stimulate delta cells to secrete somatostatin, which inhibits both insulin and glucagon secretion and thus may serve as an additional term implementing the insulin inhibition on glucagon. Our model can be readily modified to include the effect of somatostatin by adding a new dynamic variable representing the levels of this hormone and updating Equations (2 and 3) with the appropriate inhibitory terms. In addition, our study considered the time-averaged secretion and ignored the pulsatile nature of insulin and glucagon. It will be interesting to extend our approach to study these additional layers of regulation. Diabetes entails a breakdown of glucose homeostasis, with glucose levels exceeding the normal range for extended periods. Our study suggests a novel feature that can give rise to transient glucose overshoots - a breakdown of the paradoxical activation of insulin secretion by glucagon. It will be important to test this prediction in an *in-vivo* model system where this interaction is perturbed^24^.

## Methods

### Parameter estimation

In this section we explain our approach for determining bounds on the key parameters in our simulations. Our estimate of the insulin sensitivity *V* placed specific constraints on the range of *K* (Equation (3)), the impact of glucose on insulin secretion, to ensure that blood glucose levels will be responsive to insulin increase. To obtain such range, we considered the case of a positive input (*INPUT* > 0), e.g. a meal and consequently neglected the effects of glucagon as well as the hepatic glucose output (*f*([*GLG*], [*INS*]) = 0). This simplifying case yields the following equations:

11 $$\frac{d[BG]}{dt}=INPUT+{\beta }_{0}-({\delta }_{b}+{\delta }_{b}DROP)[BG]-V[Rins][BG]$$

12 $$\frac{d[INS]}{dt}=\mu +K{([BG]-B{G}^{\ast })}^{+}-{\delta }_{i}INS-\varepsilon ([INS]-[Rins])$$

13 $$\frac{d[Rins]}{dt}=\varepsilon ([INS]-[Rins])-{\delta }_{Ri}[Rins].$$

In order to guarantee its contribution to the reduction of blood glucose deviations, *V*[*Rins*][*BG*] needs to be of the order of the increase in [*BG*] following a typical *INPUT*. We varied *K*, the only free parameter in the system (11–13), given our estimates of Table 1, and found that *K* needs to be larger than 10^−7^ *min*^−1^ for this symplified model. Similarly, to obtain a range for the effects of [*BG*] on glucagon we considered glucose drops. In this case, we considered Equation (2) with no paracrine interactions and without insulin action and we found *V*_*r*_ to be at least 10^−7^ *min*^−1^ for the system to be responsive. *α*, *β*_0_ and *ε* were estimated by computing steady state conditions in the case of a normal fasting person (*INPUT* = *DROP* = 0), after requiring steady state levels of blood insulin and glucagon to be 174 *pmol*/*L* and 17.2 *pmol*/*L* respectively^58,59^. In particular, ${\beta }_{0}=B{G}^{\ast }({\delta }_{b}+VRin{s}^{\ast }),$, $\alpha ={(GL{G}^{\ast }{\delta }_{g}-{I}_{g}\ast IN{S}^{\ast })}^{+}$, $\varepsilon =\frac{{\delta }_{Ri}Rin{s}^{\ast }}{IN{S}^{\ast }-Rin{s}^{\ast }}$, $Rin{s}^{\ast }=\frac{\mu -{\delta }_{i}IN{S}^{\ast }+{G}_{i}GL{G}^{\ast }}{{\delta }_{Ri}}$, *μ* was estimated by 62 *pmol*/(*min*^−1^ *L*) considering that 50 *IU* of insulin are secreted per day into a 4*L* pool of blood^4^. We estimated *V* to be 0.38 × 10^−6^ *pmol*^−1^/*min*^−1^ *L*^27^.

Simulations in the main text were performed with linear terms for the paracrine interactions: *g*([*INS*]) = *INS* and *h*([*GLG*]) = [*GLG*]. All the topologies were identified by the pair (*I*_*g*_, *G*_*i*_) where the coordinates are positive, negative or zero depending on the simulated system. Results are valid also with nonlinear interactions, modeled with the following functions:*g*([*INS*]) = ([*INS*] − INS^*^)^+^, *h*([*GLG*]) = ([*GLG*] − GLG^*^)^+^.

### Local analysis parameters

Parameters used for the simulation are *ω* = 1, *I*_*g*_ = *G*_*i*_ = 10^−5^ *min*^−1^.

### Global analysis procedure

Simulations were performed in MATLAB by varying *I*_*g*_, *G*_*i*_ separately in one of the intervals [−0.1, 0], [0, 0.1] obtaining the nine different topologies. We simulated the system 2000 times for each choice of the intervals for the pair *I*_*g*_, *G*_*i*_ and choosing randomly the set of the 3 parameters: for each choice we collected the values of the three performance criteria we considered before. Parameters *V*_*r*_ and *K* were allowed to vary in the intervals [10^−7^, 10^−3^], *ω* in interval [0, 2].

Data represented in Fig. 3e are obtained by interpolation from^37^. For each global analysis performed (Figs 3 and S3, S4 and S5), the statistical significance has been studied used the Kruskal-Wallis analysis in order to test whether the performance criteria for the 8 networks were sampled from the same distribution, obtaining always *p* < 0.001.

### Response to protein meals

To assess the perfomance of the system in Equations (7 and 8), we require that the steady states of both hormones increase upon an input (*I*_*AAg*_, *I*_*AAi*_ > 0). The nullclines of Equations (7 and 8) are given by

14 $$[GLG]=\frac{\alpha +{I}_{AAg}}{{\delta }_{g}}+\frac{g([INS])}{{\delta }_{g}}$$

15 $$INS=\frac{\mu +{I}_{AAi}}{{\delta }_{i}}+\frac{h([GLG])}{{\delta }_{i}}.$$

Equilibria (GLG^*^, INS^*^) in the case *I*_*AAg*_ = *I*_*AAi*_ = 0 and (GLG^*I^, INS^*I^) in the case with *I*_*AAg*_, *I*_*AAi*_ > 0 are given respectively by

16 $$GL{G}^{\ast }=\frac{\alpha }{{\delta }_{g}}+\frac{g(IN{S}^{\ast })}{{\delta }_{g}}$$

17 $$IN{S}^{\ast }=\frac{\mu }{{\delta }_{i}}+\frac{h(GL{G}^{\ast })}{{\delta }_{i}},$$

and

18 $$GL{G}^{\ast I}=\frac{\alpha +{I}_{AAg}}{{\delta }_{g}}+\frac{g(IN{S}^{\ast I})}{{\delta }_{g}}$$

19 $$IN{S}^{\ast I}=\frac{\mu +{I}_{AAi}}{{\delta }_{i}}+\frac{h(GL{G}^{\ast I})}{{\delta }_{i}}.$$

and our requirement for a coordinated secretion in response to an AA input is

20 $$GL{G}^{\ast I}\ge GL{G}^{\ast }$$

21 $$IN{S}^{\ast I}\ge IN{S}^{\ast }.$$

By inserting Equations (16–19) into (20 and 21) we obtain that the following constraints need to be satisfied for coordinated secretion :

22 $${I}_{AAg}\ge g(IN{S}^{\ast })-g(IN{S}^{\ast I})$$

23 $${I}_{AAi}\ge h(GL{G}^{\ast })-h(GL{G}^{\ast I}).$$

As a result, in the case of a double positive feedback (*T*_4_, Fig. 4d), Equations (22) and (23) are always satisfied since *g* and *h* are both increasing functions, and *I*_*AAg*_, *I*_*AAi*_ both positive constants.

In the case of a double negative feedback (*T*_2_, Fig. 4e), *g* and *h* are decreasing functions. Therefore, obtaining the requirements of coordinated secretion (Equations (22) and (23)) implies:

24 $$g(IN{S}^{\ast })-g(IN{S}^{\ast I})\ge 0$$

25 $$h(GL{G}^{\ast })-h(GL{G}^{\ast I})\ge 0$$

and, consequently conditions (22) and (23) will only be satisfied at certain dependencies of the production of *GLG* and *INS* on *I*_*AAg*_ and *I*_*AAi*_ have to be satisfied. As a consequence, the same result is valid in the case of a single positive interaction between hormones (*T*_7_ and *T*_8_), since in this case *g* or *h* is an increasing function.

For the negative feedback circuits (*T*_1_ and *T*_3_, Fig. 4f), *h* is increasing, thus

$$h(GL{G}^{\ast })-h(GL{G}^{\ast I})\le 0$$

and Equation (23) is always satisfied. Equation (22) will only be satisfied for certain values of *I*_*AAg*_(*AA*). As a consequence, the same result is valid in the case of a single negative interaction between hormones (*T*_5_ and *T*_6_), since in this case *g* or *h* is a decreasing function.

Thus, a positive feedback consisting of two positive interactions as well as *T*_1_, facilitates coordinated secretion for a wider range of functional dependencies of the hormone on the AA input (*I*_*AAg*_, *I*_*AAi*_) compared to the double negative circuit *T*_2_.

### Estimating the delay of remote insulin

In order to demonstrate the effect of remote insulin on dampening blood glucose overshoot after a drop, we monitored the value of this overshoot as function of the delay between insulin and remote insulin. The overshoot of blood glucose during the reversion after a drop is at its lowest level when the delay of remote insulin action is between 15–40 minutes, as shown in Supplementary Fig. 1a. At delay times longer than 40 minutes, blood glucose levels already ramp up to high level before remote insulin begin to counteract this increase. In order to change the delay of insulin, we considered a time constant *τ* on remote insulin equation, thus Equation (4) becoming

26 $$\frac{d[Rins]}{dt}=\tau (\varepsilon ([INS]-[Rins])-{\delta }_{Ri}[Rins]).$$

Varying *τ*, the delay varies and the correspondence between these two constant is shown in Supplementary Fig. S1b. Since *τ* multiplies all the vectorial field of the Equation (4), it does not affect its steady state obtained posing it equal to zero.

## Electronic supplementary material

Supplementary Information
