# Supplementary material for: Design principles of the paradoxical feedback between pancreatic alpha and beta cells
Source: Sci Rep. 2018 Jul 16;8:10694. doi: 10.1038/s41598-018-29084-4 (PMC6048053; doi:10.1038/s41598-018-29084-4)
Supplement: Supplementary file 1 — Supplementary Information [file 41598_2018_29084_MOESM1_ESM.pdf]

Supplementary Information
Design principles of the paradoxical feedback between pancreatic alpha and beta cells

Immacolata Garzilli[a], Shalev Itzkovitz[a]

[a] *Weizmann Institute of Science,Department of Molecular Cell Biology, Wolfson Building, 234 Herzl St., Rehovot 76100 Israel*

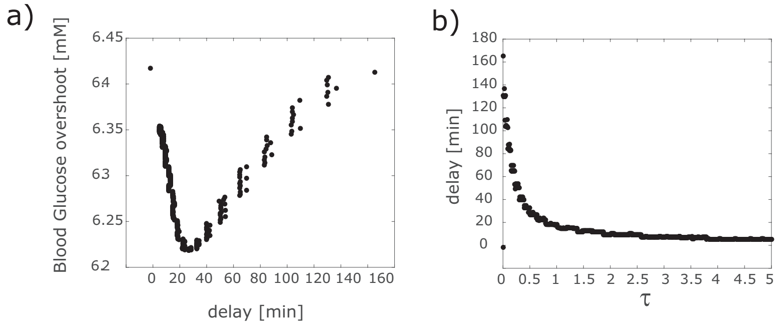

Figure S1: **Effect of remote insulin delay effect.** a) Blood glucose overshoot as function of remote insulin delay with respect to insulin; at delay times longer than 40 minutes, blood glucose levels already ramp up to high level before remote insulin begin to counteract this increase. b) Delay between remote insulin and insulin as a function of the time-constant τ, Equation (26). Parameters for simulations are reported in Table 1.

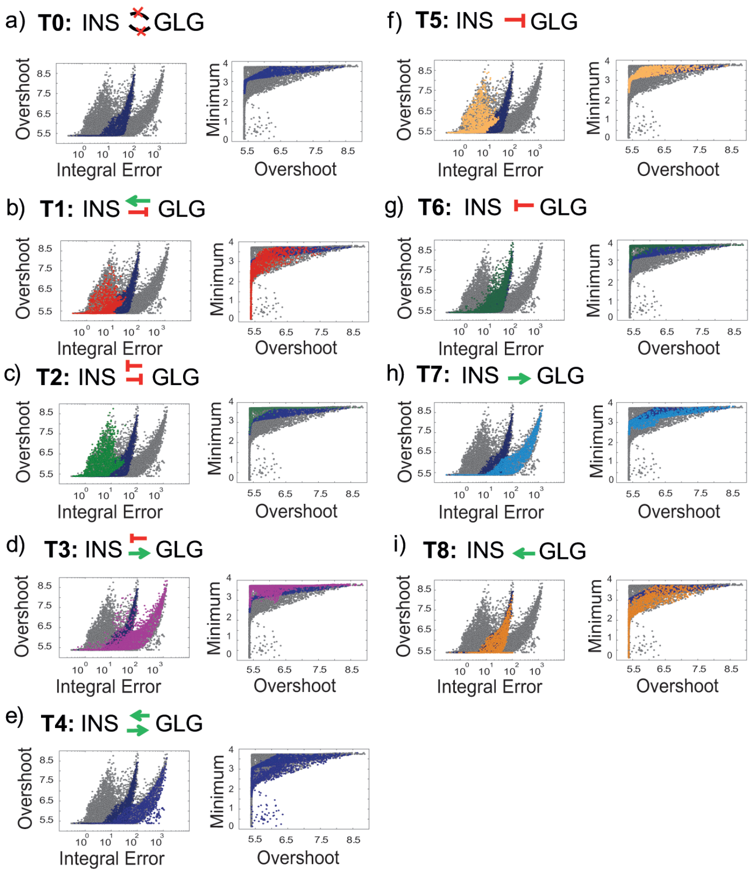

Figure S2: **Global analysis - scatter plot.** For each circuit, two plots are shown, one for the Integral Positive Error vs the Maximum after Drop, another for the Maximum vs the Minimum after Drop. For each plot, 2000 dots are shown, representing the result of a simulation with a random set of parameters chosen in the intervals defined in Table 1. For each circuit, results are compared with results of topology T_0 (blue dots) and the cumulative result from the other topologies (grey dots). Results are for T_0 (blue dots), T_1 (red dots), T_2 (green dots), T_3 (magenta dots), T_4 (purple dots), T_5 (yellow dots), T_6 (dark dots), T_7 (cyan dots), T_8 (orange dots). Units are mM for overshoots and undershoots, mM $\times$ minutes for the integral error evaluated on a 500-minute time interval.

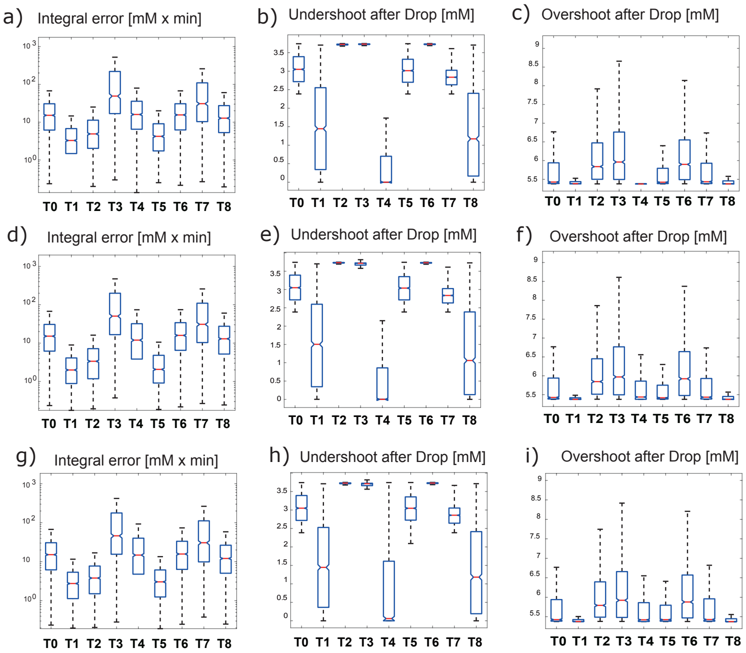

Figure S3: **Global analysis for nonlinear paracrine interactions.** Boxplots in (a),(b),(c) represent respectively the integral positive error, the minimum after drop, and the overshoot of all circuit topologies for a nonlinear paracrine interaction for both insulin and glucagon. Boxplots in (d),(e),(f) represent respectively the integral positive error, the minimum after drop, and the overshoot of all circuit topologies for a nonlinear interaction of insulin on glucagon. Boxplots in (g),(h),(i) represent respectively the integral positive error, the minimum after drop, and the overshoot of all circuit topologies for a nonlinear interaction of glucagon on insulin. Nonlinear interactions have been modeled as $(x - x^*)^+$ or $(x^* - x)^+$ depending on the case of an activator or inhibitor, where x^* represents the steady state and $(x)^+$ is 0 when $x \leq 0$ and x when $x > 0$. The variable x can be INS or GLG depending on the circuit topology. All the integral errors are evaluated on a 500-minute time interval. For each index, Kruskal-Wallis analysis reports $pvalue < 0.001$.

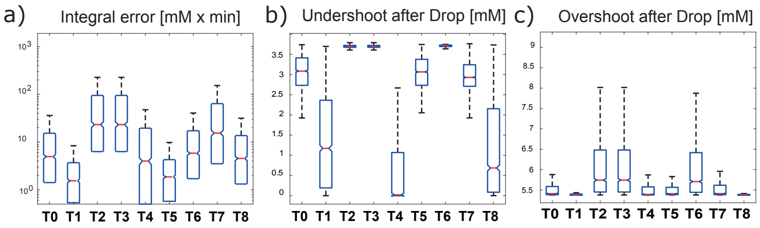

Figure S4: **Global analysis considering glycogenesis effect.** Boxplots represent the integral positive error (a), minimum after drop (b) and overshoot (c) of all circuit topologies in the case in which the term of glycogenesis $-V_g[INS][BG]$ is inserted in blood glucose equation (Equation 1) with $V_g \in [10^{-9}, 10^{-3}]$. All the integral errors are evaluated on a 500-minute time interval. For each index, Kruskal-Wallis analysis reports $pvalue < 0.001$.

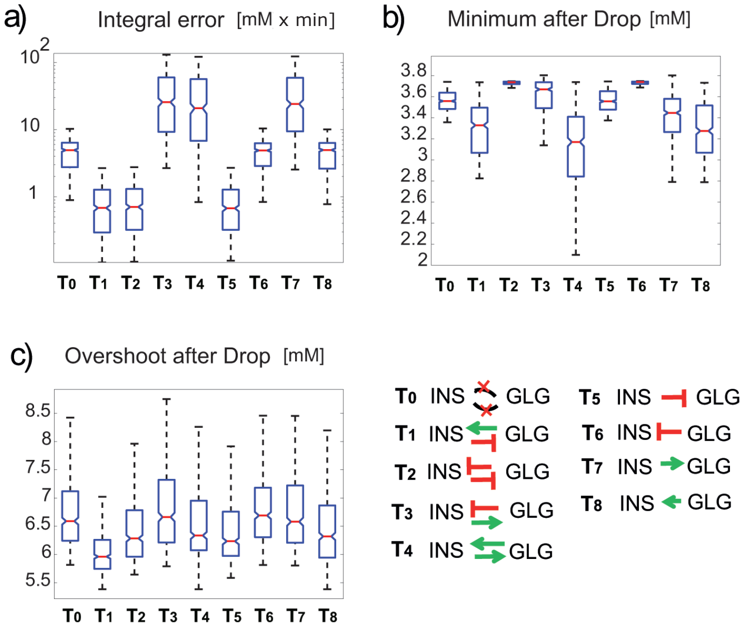

Figure S5: **Global analysis with $\omega = 1.5$.** Boxplots represent the integral positive error (a), the minimum after drop (b) and the overshoot (c) of all circuit topologies. All the integral errors have been evaluated on a 500-minute time interval. For each index, Kruskal-Wallis analysis reports $pvalue < 0.001$.

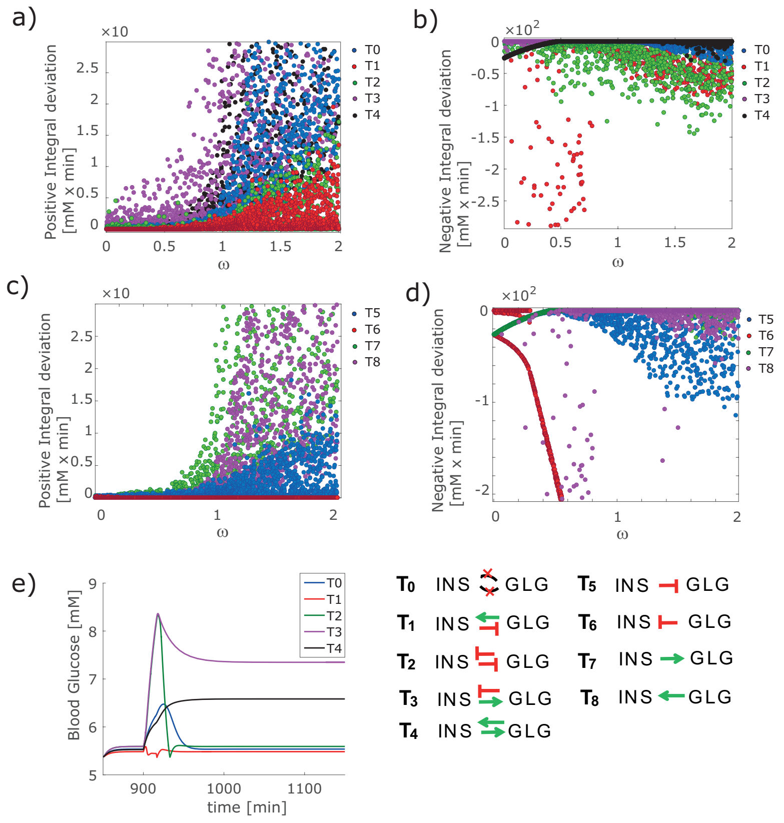

Figure S6: **Effect of an amino acid stimulus on blood glucose level.** Positive (a)-(c) and negative (b)-(d) integral deviation of blood glucose from its steady state after an amino acid stimulation on insulin and glucagon, as function of the hepatic glucose production (ω). For each plot, 2000 results are shown for topologies T0 (blue dots), T1 (red dots), T2, (green dots), T3 (purple dots), T4(black dots); e) Blood glucose level after an amino acid stimulus, acting on insulin and glucagon, for topologies T0 (blue line), T1 (red line), T2, (green line), T3 (purple line), T4(black line). Parameters are reported in Table 1 with $|Ig| = |Gi| = 0.5$; the interactions are modeled with the linear functions as in Equations (2)-(3) and INPUT and DROP are equal to 0, $\omega = 1$. In all simulations the amino acid stimulus has been provided for 30 minutes. Units are mM $\times$ minutes for both the integral errors, evaluated on a 200-minute time interval.
